# Model of Life Expectancy of Chronic Hepatitis B Carriers in an Endemic Region

**DOI:** 10.2188/jea.JE20090039

**Published:** 2009-11-05

**Authors:** Tao Wang

**Affiliations:** 1 Faculty of Health Sciences, Queen’s University, Kingston, ON, Canada

**Keywords:** hepatitis B, hepatocellular carcinoma, life expectancy, mortality, Taiwan

## Abstract

**Background:**

Chronic infection with hepatitis B (HBV) is a known risk factor for increased mortality from hepatocellular carcinoma (HCC) and chronic liver disease (CLD). However, the specific effects of chronic HBV infection on life expectancy have not been adequately studied. Taiwan is endemic for HBV infection, and thus provides sufficient information for such estimates.

**Methods:**

Population mortality statistics, combined with data on the contribution of HBV to HCC and CLD deaths, were used to model carrier mortality by sex and e antigen status. An abridged life table was used to calculate carrier life expectancy.

**Results:**

Among both males and females, those who are e antigen-positive are more likely to die from HCC than from CLD. When e antigen status remains positive, absolute liver mortality rates climb significantly after age 40 years. CLD is a proportionally higher threat for e antigen-negative females than for other subgroups. Males have higher liver-related mortality at all ages. A small decrease in life expectancy, from 82.0 to 80.1 years, was found for female noncarriers versus female carriers; a larger discrepancy was observed for males—from 76.2 to 71.8 years. In comparison to noncarriers, the lifetime relative risk of mortality is 1.35 for male carriers and 1.16 for female carriers.

**Conclusions:**

These results indicate that chronic HBV infection results in significant liver-related mortality; however, carriers retain a satisfactory life expectancy.

## INTRODUCTION

There are an estimated 350 million chronic carriers of hepatitis B virus (HBV) in the world.^1^ In endemic regions such as China and Southeast Asia, perinatal and early childhood infections are the predominant sources of chronic infection^2^ and are associated with 90% and 30% probabilities of chronicity, respectively.^3^^,^^4^ In contrast, there is only a 5% to 10% chance of developing chronic infection among people infected as adults.^3^ Earlier infection has been associated with higher rates of complications such as cirrhosis and hepatocellular carcinoma (HCC).^5^ Despite the advent of effective vaccines, many HBV-endemic countries have only recently adopted universal vaccination.^6^

Studies to date have revealed several prognostic factors for chronic carriers—including gender, age, and HbeAg status—which influence the likelihood of progression to cirrhosis or hepatocellular carcinoma.^7^^–^^9^ However, few studies have specifically examined the life expectancy of hepatitis B carriers. Although mortality models have been created, most use static values for variables such as surface antigen prevalence, e antigen status, and HBV-associated liver cancer.^10^^,^^11^ The present study aims to develop a realistic mortality model by using dynamic age- and sex-specific values. Because Taiwan is an HBV-endemic area and has been the focus of many epidemiological studies, it is possible to create a comprehensive survival model of carriers. Life expectancy and mortality data would aid clinicians in offering prognoses, which would benefit the many patients who report feelings of depression related to insufficient prognostic information.^12^ Public health epidemiologists can use this model to estimate regional disease burden and predict the results of vaccination and treatment interventions. Finally, this method of estimating carrier life expectancy can be applied to determine the cost-effectiveness of treatment and thus may potentially influence drug reimbursement schedules.

## METHODS

### Mortality data

Mortality data were obtained from Taiwan’s 2006 Statistics of Causes of Death report. These data describe liver cancer- and chronic liver disease (CLD)-related deaths for the general population, by sex and age.^13^ Because it has been reported that there were no significant differences in non-liver-related mortality between HBV carriers and noncarriers,^7^ non-liver-related deaths were calculated by subtracting mortality associated with liver cancer and chronic liver disease from overall mortality rates. The impact of HBV on infant mortality, defined as death before the age of 1 year, was not considered in this analysis, which focused instead on the effects of chronic disease. In this model, carrier infants had the same mortality as infants from the general population.

### HbsAg

Hepatitis B surface antigen (HbsAg) is a marker of current hepatitis B infection. HbsAg seropositivity on screening tests predominantly represents chronic, rather than acute, infection.^14^^,^^15^ Age group-specific HbsAg prevalence in Taiwan has been estimated by several general population-based studies. In the early 1990s, Taiwan initiated a large community-based cancer screening project that examined HbsAg prevalence among adults aged 30 to 65 years. Age-specific data were directly reported for men, and data for women were calculated by subtracting the values for men from the overall prevalence values.^7^^–^^9^ Prevalence in people over 65 years of age was kept equal to that of those aged 60 to 65 years, due to insufficient general population data from older age groups.

For people younger than 30 years, vaccination becomes a factor in surface antigen prevalence. Taiwan initiated targeted vaccination nationwide in 1984 and universal vaccination in 1986.^16^ A person born in 1986 would have been 20 years of age at the time the 2006 Statistics of Causes of Death report was completed. The best available estimates for the seroprevalence of HbsAg in young adults come from recent university-based screening programs, in which people younger than 30 years were subdivided into those younger than 20, 20 to 24, and 25 to 29 years of age. The HbsAg prevalences among adults aged 20 to 24 and 25 to 29 years were estimated by using the arithmetic means of the values for different birth years in a study conducted at Fu-Jen Catholic University, in northern Taiwan.^16^ Prevalence for those younger than 20 years was estimated at 3%, based on a nationwide study recently conducted among Taiwanese preschoolers.^17^ It is believed that the introduction of universal vaccination in 1986 led to equal seropositivity rates among all people younger than 20 years.

### HbeAg

Hepatitis B e antigen (HbeAg) is a marker of high viral replication, and is useful for evaluating prognosis and treatment.^8^ Statistics regarding e antigen prevalence were obtained from large population-based studies.^8^^,^^18^ Yang et al^8^ examined adult male carriers and provided data on e antigen prevalence among adults aged 30 to 65 years. Although a study by Chu et al^18^ investigated asymptomatic carriers only, and thus has an element of selection bias, it was nonetheless deemed appropriate to use their data on asymptomatic patients aged 15 to 29 years in the present model.^18^ Because relatively few carriers in the younger age group are symptomatic, selection bias should be minimal. The 2 data sets were combined to encompass HbeAg prevalence from age 15 to 65 years. The present model assumed equal e antigen prevalence between male and female carriers.^19^ The prevalence of e antigen among people over 65 years was held equal to that of those aged 65, due to insufficient data from older age groups.

Adult HbeAg-positive carriers have a worse prognosis, with relative risks of 6.27 and 2.2 for hepatocellular carcinoma and chronic liver disease mortality, respectively.^7^^,^^8^ HbeAg prevalence appeared to decrease exponentially, so the data were best fitted to an exponential curve using Curve Expert, a software program that employs the Levenberg–Marquardt algorithm.^20^^,^^21^ E antigen status was only taken into consideration after the age of 15 years, because the prognostic value of HbeAg is better validated in adolescents and adults.^22^ In addition, most children are in a quiescent immune-tolerant phase of the disease.^23^ The e antigen prevalence for each age interval was calculated with the curve-fitted equation using the median age of the interval.

### Hepatocellular carcinoma

Hepatitis B infection markedly increases an individual’s risk of hepatocellular carcinoma, the predominant form of liver cancer. Its effect on other forms of primary liver cancer (PLC) is less clear,^24^ although it is assumed that the incidences of non-HCC liver cancers are similar in carriers and the general population. HCC accounts for approximately 92% and 85% of all PLCs among Taiwanese men and women, respectively.^25^

The relative contribution of hepatitis B to HCC has been examined retrospectively.^26^^,^^27^ In Taiwanese younger than 40 years, approximately 90% of HCC cases were positive for HBV.^27^ In a related study, in which the vast majority of patients were older than 40 years, gender was also found to affect the prevalence of HBV in liver cancer: among Taiwanese HCC patients, 67% of men were HbsAg-positive, as compared to 41% of women.^26^ It was thus assumed in the present model that among all people younger than 40 years, regardless of sex, 90% of HCC cases were associated with hepatitis B. For those older than 40 years, the values of 67% and 41% were used for men and women, respectively.

### Chronic liver disease

The Taiwan cohort of the early 1990s demonstrated that people with hepatitis B and hepatitis C (HCV) had similar rates of CLD mortality.^28^ Based on that cohort’s data on person-years of follow-up and mortality, it is estimated that hepatitis B is associated with approximately 45% of CLD mortality in Taiwan. Since HBV generally plays a greater role in males than in females, due to its higher prevalence among the former, values of 50% and 40%, respectively, were used in the present model. Unfortunately, the dynamics of the age-related HBV contribution to CLD death are not well understood.

### Mortality and survival plots

General population mortality data were provided for age groups in 5-year intervals. Liver cancer mortality was adjusted for the proportion with an HCC diagnosis, the percentage of HCC associated with hepatitis B, and the population prevalence of HbsAg. The same process was performed for CLD death. Mortality was also analyzed with respect to HbeAg status and adjusted by relative risk and age-specific HbeAg prevalence. The overall carrier mortality rate was obtained by summing the rates of non-liver, HCC, CLD, and non-HCC liver cancer. The values for noncarriers were calculated using the same methods. Survival plots and life expectancies were obtained by using an abridged life table, whereby a hypothetical cohort was subjected to age-specific mortality rates, with survivors proceeding to the subsequent age interval.^29^ Relative mortality risks were calculated and standardized to the 2006 Taiwan population distribution.

### Sensitivity analysis

To determine the extent to which input estimation altered final life expectancy, sensitivity analysis was performed on several variables. Only one input was analyzed at a time, while the others were held at their original values. Surface antigen prevalence, HBV-associated HCC death, and the fraction of HCC within primary liver cancers were all given upper and lower estimates of 125% or 75%, respectively, of their original value. Because the data were less conclusive regarding HBV-associated CLD death, the estimates were 150% and 50% of the original estimate. In addition, upper and lower estimates for life expectancy and relative mortality risk were used, with all variables set towards highest or lowest mortality.

## RESULTS

Hepatitis B prevalence, as measured by HbsAg, continued to increase until the fourth decade, after which it declined (Table 1). Males had equal or higher seropositivity than females in all age groups. The lowest rates of infection were found in people younger than 20 years, ie, those who were born after universal vaccination was introduced.

**Table 1.** Prevalence of hepatitis B surface antigen in Taiwan, by sex and age group

Age group (yrs)	HbsAg prevalence (%)^a^	

	Male	Female^b^
<20	3	3
20–24	6.1	4.5
25–29	12.7	9.7
30–39	23.8	14.9
40–49	21.9	13.8
50–59	18.9	12.7
>60	12.5	10.7

^a^prevalence data are compiled from several population-based surveys on seroprevalence.^7^^–^^9^^,^^16^^,^^17^

^b^prevalence data for females aged 30 to 59 years were calculated by subtracting available data for males from overall prevalence, using studies based on the same cohort.^7^^–^^9^

**Figure 1.**
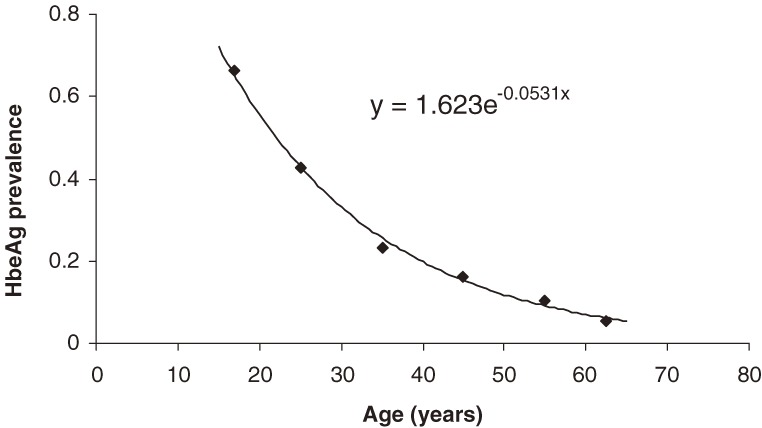
The prevalence of hepatitis B e antigen by age, based on data from 2 seroprevalence surveys.^8^^,^^18^ The data were curve fitted with Curve Expert using the Levenberg-Marquardt method.^20^^,^^21^ The equation of the curve is displayed; the correlation coefficient was 0.998.

HbeAg prevalence is the percentage of HbsAg carriers who are also HbeAg-positive. Figure 1 shows an age-specific prevalence curve that has been fitted to an exponential decay model. The percentage of e-positive carriers ranges from approximately 66% among people aged 15 to 19 years to 5% among those 65 years or older.

**Figure 2.**
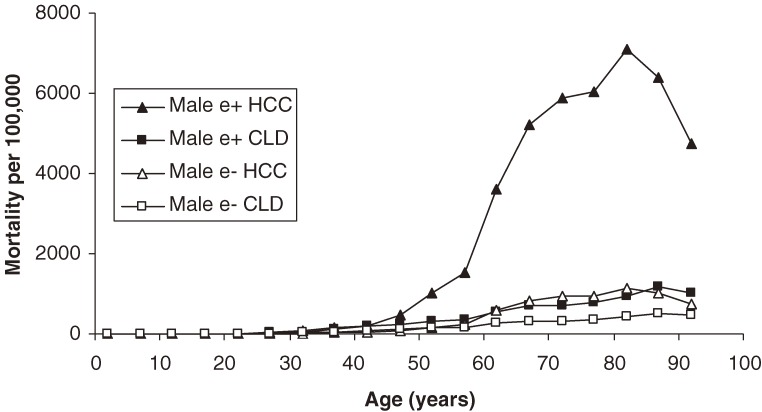
Age-specific mortality among male hepatitis B carriers caused by hepatocellular carcinoma and chronic liver disease, by e antigen status.

**Figure 3.**
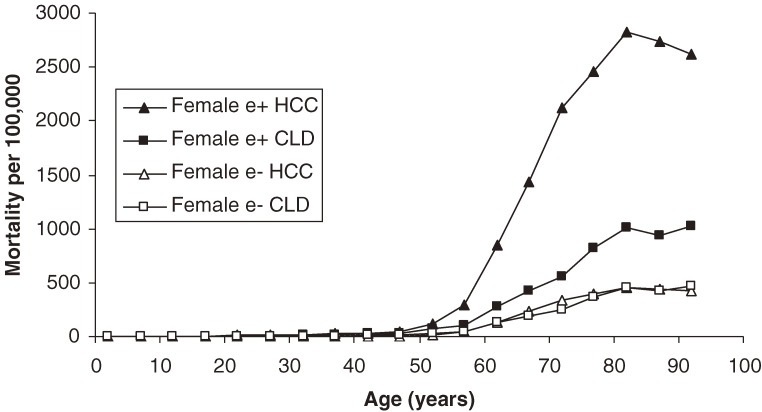
Age specific mortality among female hepatitis B carriers caused by hepatocellular carcinoma and chronic liver disease, by e antigen status.

Regarding mortality trends, e antigen-positive men are significantly more likely to die from HCC than from CLD; e antigen-negative men have less divergence between the rates for HCC and CLD (Figure 2). Among females, chronic liver disease represents a relatively higher proportion of liver-related deaths (Figure 3). Among those aged 80 to 84 years, HCC carries the highest risk among both men and women. Since e antigen conveys a 6.27 RR for HCC mortality, e-positive adults are always at higher risk. When subgrouped by sex and HbeAg status, peak HCC mortality varies greatly. For example, e antigen-positive males have a peak HCC mortality around 7000 per 10^5^. In contrast, the peak for e antigen-negative females is approximately 370 per 10^5^. Thus, there is nearly a 20-fold difference in peak HCC mortality between the highest and lowest risk carrier subpopulations. Chronic liver disease mortality decreases slightly among adults aged 85 to 89 years and is less influenced by e antigen status. There is also less of a gender gap in CLD mortality in that age group, although males still have a worse prognosis.

**Figure 4.**
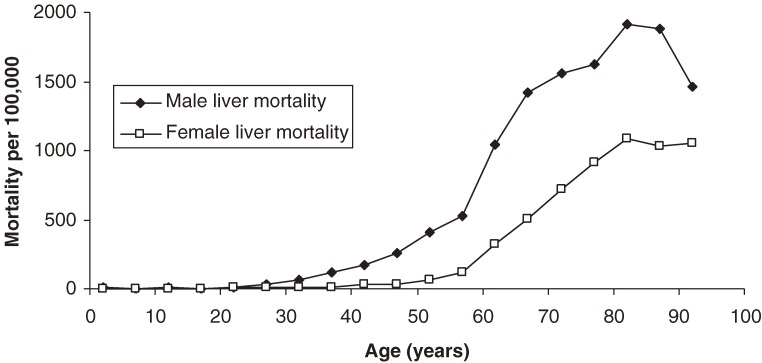
Overall liver-related age-specific mortality of hepatitis B carriers, by sex.

Figure 4 shows liver mortality rates among male and female HBV carriers. Before age 30 years, liver mortality is rare. Male carriers surpass a liver mortality rate of 100 per 10^5^ at approximately the age of 35 years, whereas female carriers do so at approximately the age of 55 years. By age 60 years, males have a liver mortality rate of 1000 per 10^5^; female carriers on average never reach this rate. Adults aged 80 to 84 years have the highest liver mortality: 1900 and 900 per 10^5^ in men and women, respectively. The graph illustrates that as age increases the ratio of male to female deaths decreases but the absolute differences do not.

Male carriers have an overall relative risk (RR) of death of 1.35, as compared to noncarriers aged 1 to 89 years, standardized to the 2006 Taiwan population. Female carriers have an RR of 1.16.

**Figure 5.**
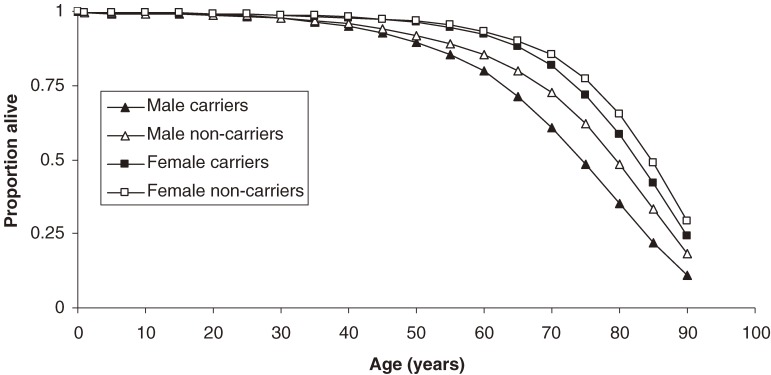
Survival of hepatitis B carriers versus noncarriers, by sex.

The survival plot (Figure 5) shows that female HBV carriers experience a modest reduction in life expectancy, which shifts the mean life expectancy from 82.0 to 80.1 years. Among men, the effect is more pronounced, resulting in a loss of 4.4 years—from 76.2 to 71.8 years. The 75% survival mark is reached at age 62.8 years for male carriers, versus age 68.3 years in noncarriers. Among women, 75% of carriers survive to age 73.4 years, while 75% of noncarriers live to be 76.0 years.

Sensitivity analysis was performed to test the impact of altering surface antigen prevalence, the percentage of HBV-associated HCC, the percentage of HCC among primary liver cancers, and the percentage of HBV-associated CLD (Table 2). The analysis revealed that the output is most sensitive to alterations in HbsAg prevalence. However, the differences between the high and low estimates are all less than 2.3 years. Table 3 shows outcomes when all variables in sensitivity analysis are shifted in favor of or against survival. In this scenario, the low and high estimates vary by only 3.2 years for women, in comparison to 5.9 years for men. Based on the extremes of sensitivity analysis, the estimated relative mortality risks would range from 1.09 to 1.74 for men and 1.04 to 1.34 for women. All input changes produce greater output variations in males than in females.

**Table 2.** Sensitivity analysis of the life expectancy of hepatitis B carriers after alteration of input variables

Input Variables^a^	Life expectancy (years)	

	Male	Female
HbsAg prevalence		
​ Low	70.4	79.4
​ High	72.7	80.5
HBV-associated HCC		
​ Low	72.5	80.4
​ High	71.1	80.0
HBV-associated CLD		
​ Low	72.5	80.5
​ High	71.0	79.6
%HCC in PLC		
​ Low	72.3	80.3
​ High	71.6	79.9

^a^The analyzed input was varied between a low and a high estimate, while all other variables were held at the original estimate. Low and high estimates represent 75% and 125% of the original input, respectively; however, for HBV-associated CLD, the low and high estimates are 50% and 150% of the original input.

**Table 3.** Low, middle, and high estimates for life expectancy among hepatitis B carriers, as compared to noncarriers

	Life expectancy (years)	
	
	Male	Female
Carrier^a^		
Low	68.4	78.1
Middle	71.8	80.1
High	74.3	81.3
Noncarrier	76.2	82.0

^a^Low and high estimates were calculated by aligning all 4 sensitivity analysis variables from Table 2 against or in favor of survival, respectively. The middle value is the original estimate for life expectancy.

## DISCUSSION

Chronic hepatitis B is a common disease in Taiwan, affecting 15% to 20% of the general population.^7^^,^^8^ Universal vaccination has had a very positive impact among the younger population, but most people over 20 years of age have not been vaccinated. Thus, chronic liver disease is expected to remain a major health concern in Taiwan for many decades. As shown in Table 1, the highest prevalence of surface antigen is found in adults aged 30 to 39 years. This may simply be a cohort effect, but it may also reflect the combined effects of chronic infection early in life and horizontal transmission as people age. The subsequent decline in prevalence may reflect the combined effects of higher mortality amongst carriers and HbsAg seroclearance.

The exponentially decreasing prevalence of HbeAg results from seroconversion caused by an active immune response against infected hepatocytes.^23^ Seroconversion tends to occur later in the course of infection, which results in a lower prevalence of HbeAg among older age groups (Figure 1).^8^ As shown in Figures 2 and 3, e antigen-positive individuals have considerably higher liver mortality, particularly for HCC. It is important to note, however, that the majority of e antigen-positive individuals are in a younger age group, in which absolute mortality rates are low. Once an individual passes the age of 40 years, the consequences of e antigen positivity are more significant, due to higher baseline risks. Delayed e antigen seroconversion to anti-Hbe, such as e antigen positivity after the age of 40 years, has been associated with poor prognosis.^23^ Despite relatively low liver mortality in people younger than 40 years, the development of cirrhosis can precede CLD death by many years.^30^ Thus, a carrier who dies at 40 years of age may have already developed cirrhosis in their fourth decade of life.

HCC is more heavily implicated in male, as compared to female, mortality, regardless of e antigen status (Figures 2 and 3). It is well established that HCC is a predominantly male phenomenon, with a reported male to female ratio ranging from 2:1 to 7:1.^26^^,^^31^^,^^32^ It has been hypothesized that hormonal influences play a major role, and the fact that the male to female mortality ratio appears to decrease after menopause supports this hypothesis (Figure 4). Although female carriers never reach the same absolute mortality rates as males, the liver mortality slopes become nearly parallel, beginning at approximately age 55 years (Figure 4). Unfortunately, relatively few human studies have examined hormonal factors and thus their precise role is not known.^33^ Further study of these variables may yield more potential risk factors and treatments.

Figure 4 shows that men surpass a liver mortality rate of 100 per 10^5^ at approximately age 35 years; women reach this mortality rate at approximately age 55 years. Based on these findings, it would be advisable to regularly screen carriers for HCC and cirrhosis at least 5 to 10 years before these ages.

In males, the relative risk of mortality is estimated at 1.35 (1.09–1.74), as compared to noncarriers. The estimated carrier life expectancy is 71.8 years, as compared to 76.2 years among noncarriers (Figure 5). These results are consistent with other estimates, which indicate that 15% to 40% of HBV carriers die of liver complications.^34^ Although an individual carrier’s risk of complications rises with increasing age, a sizable proportion of HCC and CLD deaths is distributed among men younger than 60 years.^26^ This can be explained by the demographic distribution in Taiwan: the number of men aged 40 to 59 years is more than 2.6 times that of men aged 60 to 79 years.^13^

Despite a better prognosis, the gravity of hepatitis B in females must not be dismissed. Among women, chronic HBV infection increases the relative risk of mortality to 1.16 (1.04–1.34), as compared to noncarriers, which is consistent with the findings of previous research.^34^ This results in a decrease in life expectancy from 82.0 years in noncarriers to 80.1 years in carriers (Figure 5). Thus, the risk of liver-related mortality in female carriers is nearly 3 times higher than that of breast cancer, a disease commonly feared by women.^35^ In addition, females are able to transmit the causative virus vertically; thus, proper medical management is essential for both individual and public health.

Sensitivity analysis (Table 2) showed that the model is most sensitive to variability in HbsAg prevalence. However, variations in any single variable had a minimal impact on estimates. Although liver disease is one of the highest specific causes of mortality in carriers, the majority of carrier deaths are not liver-related. Therefore, changes in single input variables have only a modest impact on overall life expectancy. In Table 3, in which all factors are aligned in favor of or against survival, the high and low estimates are only 3.2 years apart for women. For men the estimate range is higher—5.9 years. These differences in range are the result of greater liver mortality in men, ie, changes in percentage input have a greater effect on absolute output values.

The results of this model are consistent with those of Pokorski and Ohlmer.^36^ Using a Markov model, they estimated mortality risk in Asian carriers to be between 1.50 and 1.75 for men and 1.25 and 1.50 for women. Cohort studies from Australia and Taiwan have also yielded similar results: 1.4 (1.3–1.5) and 1.7 (1.5–1.9), respectively.^7^^,^^37^ The present model produced lower relative mortality estimates than did the other studies, perhaps because the cohort studies were restricted to middle-aged and young-old adults.

There are some limitations in this study. The role of treatment using interferon and antivirals was not considered in the present model. These treatments, however, were not reimbursed by the Taiwanese government until 2003 and thus have not been widely used.^34^

Seroclearance of HbsAg is a modifier of surface antigen prevalence when chronic carriers were followed over decades.^38^ Chronic carriers who clear HbsAg generally have good prognoses, but nevertheless remain at greater risk than the general population.^39^ Only a small number of studies have been conducted to determine the rate of clearance. Not accounting for these former carriers causes a slight overestimation of carrier risk.

The status of HbeAg-negative chronic active hepatitis cannot be accommodated in this analysis due to its unconfirmed prevalence in Taiwan. In addition, the definition of e-negative chronic active hepatitis is still debated, with questions surrounding the adequacy of a DNA level cut-off versus the need for biopsy confirmation.^40^ The prevalence of e-negative active disease also varies with age; however, no age-specific data exist.^41^

Although HBV DNA is a known prognostic factor for chronic carriers, it is a highly dynamic variable and studies with regular follow-up and repeated DNA measurements are lacking.^34^ Until such studies become available, this factor cannot be reliably modeled.

Confidence intervals were not provided in this model due to the use of data derived from studies involving large sample sizes. The standard errors of these studies would thus have played a minor role in causing estimation error in comparison to the variation of inputs, as demonstrated in the sensitivity analysis.

An advantage of a mathematical model derived from population data is the relative avoidance of the selection bias that is inevitably present in tertiary-center studies. It also avoids the bias of research conducted solely on asymptomatic carriers. The present model will aid clinicians in providing information on prognosis, but it can also be employed in public health or economics research on hepatitis B disease burden and cost-benefit analysis.

The present model indicates that hepatitis B carriers in Taiwan have a reasonable life expectancy. With proper medical management and the advent of new antivirals, the prognosis for individual carriers will likely improve in the near future. The overall health burden of this disease, however, is likely to remain high in endemic areas for many decades to come.

## APPENDIX

### HbsAg

Male carrier rates are reported for all age ranges.^8^ Female HbsAg carrier rates between the ages of 30 and 65 years are calculated by subtracting male values from overall values.^7^^,^^9^

$$P_{\mathrm{bf}}=(n_{\mathrm{bt}}-n_{\mathrm{bm}})/((n_{\mathrm{bt}}+n_{\mathrm{ct}}+n_{\mathrm{nt}})-(n_{m}))$$

Where P_bf_ is the female %prevalence of HBV; n_bt_ is the total number of HbsAg-positive patients; n_bm_ is the number of male HbsAg-positive patients; n_ct_ is the total number of anti-HCV-positive but HbsAg-negative patients; and n_nt_ is the total number of HbsAg- and anti-HCV-negative patients.

### Mortality

Mortality calculations were performed for carriers and noncarriers. Unless otherwise noted, all variables represent a specific age group and sex.

**Step 1:** Calculate HCC and non-HCC PLC mortality rates.

$$\begin{array}{l} \mathrm{For carriers}:\;m_{\mathrm{hc}}=(m_{l}\times L_{h})\times H_{b}/P_{s}\\ \mathrm{For noncarriers}:\;m_{\mathrm{hn}}=(m_{l}\times L_{h})\times (1-H_{b})/(1-P_{s})\\ \mathrm{Non-HCC PLC mortality}:\;m_{n}=m_{l}\times (1-L_{h}) \end{array}$$

Where m_hc_ is the carrier HCC mortality rate (MR); m_l_ is the MR of all liver cancers in the general population; L_h_ is the % of PLC diagnosed as HCC; H_b_ is the %prevalence of HBV in HCC cases; P_s_ is the %prevalence of HbsAg in the population; m_hn_ is the noncarrier HCC MR; m_n_ is the MR of non-HCC liver cancers in both carriers and noncarriers.

**Step 2:** Calculate CLD mortality rates.

$$\begin{array}{l} \mathrm{For carriers:}\;m_{\mathrm{cc}}=m_{\mathrm{cg}}\times C_{b}/P_{s}\\ \mathrm{For noncarriers:}\;m_{\mathrm{cn}}=m_{\mathrm{cg}}\times (1-C_{b})/P_{s} \end{array}$$

Where m_cc_ is the carrier CLD MR; m_cg_ is the MR of CLD in general population; C_b_ is the % of CLD deaths associated with HBV; m_cn_ is the noncarrier CLD MR.

**Step 3:** Perform separate calculations for e-positive and e-negative HBV patients

$$\begin{array}{l} E_{\mathrm{hp}}=R_{h}\times m_{\mathrm{hc}}/((R_{h}-1)\times P_{e}+1)\\ E_{\mathrm{cp}}=R_{c}\times m_{\mathrm{cc}}/((R_{c}-1)\times P_{e}+1)\\ E_{\mathrm{hn}}=E_{\mathrm{hp}}/R_{h}\\ E_{\mathrm{cn}}=E_{\mathrm{cp}}/R_{c} \end{array}$$

Where E_hp_ is the HCC MR in e-positive individuals; E_cp_ is the CLD MR in e-positive individuals; E_hn_ is the HCC MR in e-negative individuals; E_cn_ is the CLD MR in e-negative individuals; R_h_ is the relative risk of e positivity for HCC; R_c_ is the relative risk of e positivity for CLD; P_e_ is the %prevalence of e antigen in carriers.

**Step 4:** Calculate total mortality by summing mortality causes. The sample calculation for carriers is shown.

$$M_{c}=m_{\mathrm{hc}}+m_{\mathrm{cc}}+m_{n}+m_{o}$$

Where M_c_ is the overall carrier MR; m_o_ is the total non-liver related MR of the general population.

### Relative risk

The relative risks are calculated by standardizing mortality rates in each age group with the 2006 population demographics of Taiwan.

$$\mathrm{RR}=\sum_{i=1}^{n}\left(M_{\mathrm{ci}}/M_{\mathrm{ni}}\times A_{i}/T\right)$$

Where i = 1 represents those aged 1–4 years, 2 those aged 5–9 years…up to those aged 85–89 years; RR is the relative risk of mortality; M_ci_ is the MR of carriers in the i^th^ age interval; M_ni_ is the MR of noncarriers in the i^th^ age interval; A_i_ is the population size of the i^th^ age intervals; T is the total population of Taiwan aged from 1 to 89 years in 2006.

### Survival curve

An abridged life table was used to calculate survival.^29^ An initial hypothetical cohort of 100 000 people starting at age 0 is assumed to experience the same mortality rates for the duration of each age interval. The survivors then proceed to experience the mortality of the subsequent interval.

**Step 1:** Calculate the probability of dying between the ages x and x + n, where n is the length of the age interval.

$${}_{n}q_{x}=(n\times_{n}M_{x})/(1+n\times (1{-}_{n}a_{x})\times_{n}M_{x})$$

Where _n_q_x_ is the probability of dying between the ages x and x + n; _n_M_x_ is the MR between ages x and x + n; _n_a_x_ is the estimated fraction of the age interval experienced by individuals who die between the ages of x and x + n, where _1_a_0_ = 0.1, _4_a_1_ = 0.4 and all other _n_a_x_ = 0.5.

**Step 2:** Calculate the number of people alive at each age and the number who die in each interval

$$\begin{array}{l} I_{x+n}=I_{x}\times (1{-}_{n}q_{x})\\ {}_{n}d_{x}=I_{x}-I_{x+n} \end{array}$$

For the last open-ended age interval (age 90+ years), d_90+_ = I_90_, because all remaining people will die eventually.

Where I_x_ is the number of people alive at age x, with I_0_ as the initial cohort of 100 000; _n_d_x_ is the number of people who die between ages x and x + n.

**Step 3:** Calculate the total number of person-years lived contributed by those alive and those who died between the ages of x and x + n

$$\begin{array}{l} {}_{1}L_{0}=I_{1}+0.1\times_{1}d_{0}\\ {}_{4}L_{1}=4\times I_{5}+4\times (0.4\times_{4}d_{1})\\ {}_{n}L_{x}=n\times (I_{x+n}+I_{x})/2 \end{array}$$

Where _n_L_x_ is the total number of person-years lived between the ages of x and x + n.

**Step 4:** Calculate life expectancy

$$e=\sum {}_{n}L_{x}/I_{0}$$

Where e is the life expectancy.

## References

[1] Lee WM Hepatitis B virus infection . N Engl J Med. 1997;337:1733–45 10.1056/NEJM199712113372406 9392700

[2] Beasley RP Hepatitis B virus: the major etiology of hepatocellular carcinoma . Cancer. 1988;61:1942–56 10.1002/1097-0142(19880515)61:10<1942::AID-CNCR2820611003>3.0.CO;2-J 2834034

[3] McMahon BJ , Alward WL , Hall DB , Heyward WL , Bender TR , Francis DP , Acute hepatitis B virus infection: relation of age to the clinical expression of disease and subsequent development of the carrier state . J Infect Dis. 1985;151:599–603 3973412 10.1093/infdis/151.4.599

[4] Chang MH Natural history of hepatitis B virus infection in children . J Gastroenterol Hepatol. 2000;15(5 Suppl 1):E16–9 10.1046/j.1440-1746.2000.02096.x 10921376

[5] Chu CM Natural history of chronic hepatitis B virus infection in adults with emphasis on the occurrence of cirrhosis and hepatocellular carcinoma . J Gastroenterol Hepatol. 2000;15(5 suppl 1):E25–30 10.1046/j.1440-1746.2000.02097.x 10921378

[6] Centers for Disease Control and Prevention Global progress toward universal childhood hepatitis B vaccination . MMWR Morb Mortal Wkly Rep. 2003;52:868–70 12970620

[7] Iloeje UH , Yang HI , Jen CL , Su J , Wang LY , You SL , Risk and predictors of mortality associated with chronic hepatitis B infection . Clin Gastroenterol Hepatol. 2007;5:921–31 10.1016/j.cgh.2007.06.015 17678844

[8] Yang HI , Lu SN , Liaw YF , You SL , Sun CA , Wang LY , Hepatitis B e antigen and the risk of hepatocellular carcinoma . N Engl J Med. 2002;347(3):168–74 10.1056/NEJMoa013215 12124405

[9] Chen CL , Yang HI , Yang WS , Liu CJ , Chen PJ , You SL , Metabolic factors and risk of hepatocellular carcinoma by chronic hepatitis B/C infection: a follow-up study in Taiwan . Gastroenterology. 2008;135:111–21 10.1053/j.gastro.2008.03.073 18505690

[10] Dickinson JA , Wun YT , Wong SL Modelling death rates for carriers of hepatitis B . Epidemiol Infect. 2002;128:83–92 10.1017/S0950268801006410 11895095 PMC2869799

[11] Goldstein ST , Zhou FJ , Hadler SC , Bell BP , Mast EE , Margolis HS A mathematical model to estimate global hepatitis B disease burden and vaccination impact . Int J Epidemiol. 2005;34(6):1329–39 10.1093/ije/dyi206 16249217

[12] Kunkel E , Kim JS , Hann HW , Oyesanmi O , Menefee L , Field H , Depression in Korean immigrants with hepatitis B and related liver diseases . Psychosomatics. 2000;41(6):472–80 10.1176/appi.psy.41.6.472 11110110

[13] 2006 Statistics of causes of death, Republic of China (Taiwan), 2007, Department of Health, Executive Yuan, Taiwan.

[14] Alexopoulos CG , Vaslamatzis M , Hatzidimitriou G Prevalence of hepatitis B virus marker positivity and evolution of hepatitis B virus profile, during chemotherapy, in patients with solid tumours . Br J Cancer. 1999;81(1):69–74 10.1038/sj.bjc.6690652 10487614 PMC2374347

[15] Dragosics B , Ferenci P , Hitchman E , Denk H Long-term follow-up study of asymptomatic HBsAg-positive voluntary blood donors in Austria: a clinical and histologic evaluation of 242 cases . Hepatology. 1987;7(2):302–6 10.1002/hep.1840070215 3557309

[16] Su FH , Huang HY , Chang HJ , Jen JJ , Liu YH , Chen CD Forecasting the declining rate of chronic hepatitis-B carrier status at a Taiwanese university: twenty years after implementation of an universal HBV vaccination program in Taiwan . Chang Gung Med J. 2007;30(6):521–8 18350735

[17] Lin JB , Lin DB , Chen SC , Chen PS , Chen WK Seroepidemiology of hepatitis A, B, C, and E viruses infection among preschool children in Taiwan . J Med Virol. 2006;78:18–23 10.1002/jmv.20517 16299720

[18] Chu CM , Sheen IS , Lin SM , Liaw YF Sex differences in chronic hepatitis B virus infection: studies of serum HbeAg and alanine aminotransferase levels in 10,431 asymptomatic Chinese HbsAg carriers . Clin Infect Dis. 1993;16(5):709–13 8507764 10.1093/clind/16.5.709

[19] Szmuness W , Neurath AR , Stevens CE Prevalence of hepatitis B anti-gen and its antibody in various HBsAg carrier populations . Am J Epidemiol. 1981;113:113–21 7468569 10.1093/oxfordjournals.aje.a113074

[20] Moré JJ. The Levenberg-Marquardt algorithm: Implementation and theory. In: Watson GA, editor. Numerical Analysis. Berlin: Springer-Verlag; 1978. p. 105–16. (Lecture Notes in Mathematics; vol 630).

[21] Hyams DG. CurveExpert [computer program]. Version 1.38. Hixson (TN); 2001.

[22] Sherman M , Shafran S , Burak K , Doucette K , Wong W , Girgrah N , Management of chronic hepatitis B: consensus guidelines . Can J Gastroenterol. 2007;21 Suppl C:5C–24C 17568823 PMC2794455

[23] Chu CM , Liaw YF Chronic hepatitis B virus infection acquired in childhood: special emphasis on prognostic and therapeutic implication of delayed HBeAg seroconversion . J Viral Hepat. 2007;14:147–52 10.1111/j.1365-2893.2006.00810.x 17305879

[24] Shaib Y , El-Serag HB The epidemiology of cholangiocarcinoma . Semin Liver Dis. 2004;24:115–25 10.1055/s-2004-828889 15192785

[25] Cancer Registry Annual Report, Republic of China (Taiwan), 2005, Department of Health, Executive Yuan, Taiwan.

[26] Lu SN , Su WW , Yang SS , Chang TT , Cheng KS , Wu JC , Secular trends and geographic variations of hepatitis B virus and hepatitis C virus-associated hepatocellular carcinoma in Taiwan . Int J Cancer. 2006;119:1946–52 10.1002/ijc.22045 16708389

[27] Chen CH , Chang TT , Cheng KS , Su WW , Yang SS , Lin HH , Do young hepatocellular carcinoma patients have worse prognosis? The paradox of age as prognostic factor on the survival of hepatocellular carcinoma patients . Liver Int. 2006;26:766–73 10.1111/j.1478-3231.2006.01309.x 16911457

[28] Yang HI , Chen CJ , Jen CI , Iloeje UH , Su J , You SL Causes of death associated with hepatitis B or hepatitis C virus infections in a long-term population-based cohort study. In: American association for the study of liver disease abstracts . Gastroenterology. 2007;132(4 supp 2):A-761

[29] Mathers CD, Vos T, Lopez AD, Salomon J, Ezzati M, editors. National burden of disease studies: a practical guide. 2.0 ed. Global Program on Evidence for Health Policy. Geneva: World Health Organization; 2001.

[30] Ginés P , Quintero E , Arroyo V , Terés J , Bruguera M , Rimola A , Compensated cirrhosis: natural history and prognostic factors . Hepatology. 1987;7(1):122–8 10.1002/hep.1840070124 3804191

[31] Chen PH , Lin YC , Tu HP , Chiang SL , Ko MS , Hsu CL , Important prognostic factors for the long-term survival of subjects with primary liver cancer in Taiwan: a hyperendemic area . Eur J Cancer. 2007;43:1076–84 10.1016/j.ejca.2007.01.022 17329095

[32] El-Serag HB Epidemiology of hepatocellular carcinoma . Clin Liver Dis. 2001;5(1):87–107 10.1016/S1089-3261(05)70155-0 11218921

[33] Yu MW , Chang HC , Chang SC , Liaw YF , Lin SM , Liu CJ , Role of reproductive factors in hepatocellular carcinoma: impact on hepatitis B– and C–related risk . Hepatology. 2003;38:1393–400 14647050 10.1016/j.hep.2003.09.041

[34] Chen CJ , Yang HI , Su J , Jen CL , You SL , Lu SN , Risk of hepatocellular carcinoma across a biological gradient of serum hepatitis B virus DNA level . JAMA. 2006;295(1):65–73 10.1001/jama.295.1.65 16391218

[35] Feuer EJ , Wun LM , Boring CC , Flanders WD , Timmel MJ , Tong T The Lifetime Risk of Developing Breast Cancer . J Natl Cancer Inst. 1993;85(11):892–7 10.1093/jnci/85.11.892 8492317

[36] Pokorski RJ , Ohlmer U Long-term morbidity and mortality in Chinese insurance applicants infected with the hepatitis B virus . J Insur Med. 2001;33(2):143–64 11510512

[37] Amin J , Law MG , Bartlett M , Kaldor JM , Dore GJ Causes of death after diagnosis of hepatitis B or hepatitis C infection: a large community-based linkage study . Lancet. 2006;368:938–45 10.1016/S0140-6736(06)69374-4 16962883

[38] Chu CM , Liaw YF HbsAg seroclearance in asymptomatic carriers of high endemic areas: appreciably high rates during a long-term follow-up . Hepatology. 2007;45:1187–92 10.1002/hep.21612 17465003

[39] Yuen MF , Wong DK , Sablon E , Tse E , Ng IO , Yuan HJ , HBsAg Seroclearance in Chronic Hepatitis B in the Chinese: Virological, Histological, and Clinical Aspects . Hepatology. 2004;39:1694–701 10.1002/hep.20240 15185311

[40] Al-Mahtab M , Rahman S , Khan M , Kamal M , Al Mamun A HBeAg negative chronic hepatitis B with persistently normal serum transaminase and low HBV DNA can cause significant liver disease . Indian J Gastroenterol. 2007;26:297 18431019

[41] Funk ML , Rosenberg DM , Lok AS World-wide epidemiology of HBeAg-negative chronic hepatitis B and associated precore and core promoter variants . J Viral Hepat. 2002;9:52–61 10.1046/j.1365-2893.2002.00304.x 11851903

